# Technical Note: Out‐of‐field dose measurement at near surface with plastic scintillator detector

**DOI:** 10.1120/jacmp.v17i5.6308

**Published:** 2016-09-08

**Authors:** Alexandra Bourgouin, Nicolas Varfalvy, Louis Archambault

**Affiliations:** ^1^ Département de physique de génie physique et d'optique, Université Laval Québec QC Canada; ^2^ Département de radio‐oncologie Centre Hospitalier Universitaire de Québec Québec QC Canada

**Keywords:** plastic scintillation detector, out‐of‐field dosimetry, peripheral dose, parallel‐plate ion chamber, surface dose

## Abstract

Out‐of‐field dose depends on multiple factors, making peripheral dosimetry complex. Only a few dosimeters have the required features for measuring peripheral dose. Plastic scintillator dosimeters (PSDs) offer numerous dosimetric advantages as required for out‐of‐field dosimetry. The purpose of this study is to determine the potential of using PSD as a surface peripheral dosimeter. Measurements were performed with a parallel‐plate ion chamber, a small volume ion chamber, and with a PSD. Lateral‐dose measurements (LDM) at 0.5 cm depth and depth‐dose curve (PDD) were made and compared to the dose calculation provided by a treatment planning system (TPS). This study shows that a PSD can measure a dose as low as 0.51±0.17cGy for photon beam and 0.58±0.20cGy for electron beam with a difference of 0.2 and 0.1 cGy compared to a parallel‐plate ion chamber. This study demonstrates the potential of using PSD as an out‐of‐field dosimeter since measurements with PSD avoid averaging over a too‐large depth, at 1 mm diameter, and can make precise measurement at very low dose. Also, electronic equilibrium is easier to reach with PSD due to its small sensitive volume and its water equivalence.

PACS number(s): 87.55.N, 87.55.km

## I. INTRODUCTION

**Figure 1:**
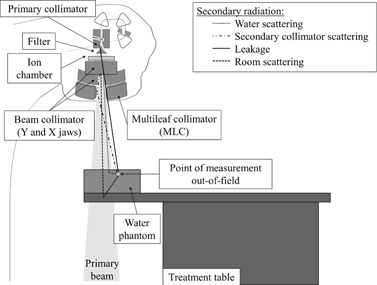
Schema of compound of the peripheral dose for external radiation therapy.

Out‐of‐field dose, or peripheral dose, is equivalent to 0.1% to 3% of the dose at $D_{\text{max}}$ in the field.^(1)^. Dose comes from water scattering, secondary collimator scattering (jaws and MLC), leakage from treatment head (interaction with primary collimator), and from room scattering.^(2)^ Out‐of‐field dose depends on multiple factors: the linac used, the energy of the primary beam, the size of the field, the depth in the phantom, and the out‐of‐field distance. Out‐of‐field dosimetry is complex, and few dosimeters have the required features for measuring peripheral dose which come from electrons and photons of high and low energy. The particles which contribute to the peripheral dose also have different angle of incidence at the point of measurement, as illustrated in Fig. 1. Out‐of‐field dosimetry requires a dosimeter with no dependence on the type of particles (electron, photon, or neutron), dose, energy, dose rate, or incidence angle. Due to the low‐energy electrons, from secondary collimator scattering, it is more difficult to reach charged‐particle equilibrium; therefore, it is preferable to have a water‐equivalent dosimeter with a small sensitive volume. Ion chambers are not equivalent to water and have usually a larger sensitive volume than other dosimeters. Diodes, liquid ion chambers, and diamond chambers depend on the dose rate.^(3)^ MOSFETs and optically stimulated luminescence (OSL) dosimeters have angular dependence.^(4)^ Plastic scintillator dosimeters (PSD) show numerous dosimetric advantages and have the required features needed for out‐of‐field dosimetry.^5^, ^6^, ^7^, ^8^ The purpose of this study is to determine the potential of using PSD as a surface peripheral dosimeter.

## II. MATERIALS AND METHODS

Measurements in this study were performed with a Varian Clinac iX (Varian Medical Systems, Palo Alto, CA). The SSD (source– surface distance) was fixed at 90 cm for photon beams and at 100 cm for electron beams except for depth‐dose‐curve measurements. The delivered dose was 200 MU at a dose rate of 600 MU/min and 400 MU/min for photon and electron beams, respectively.

### A. Dosimeter

**Figure 2:**
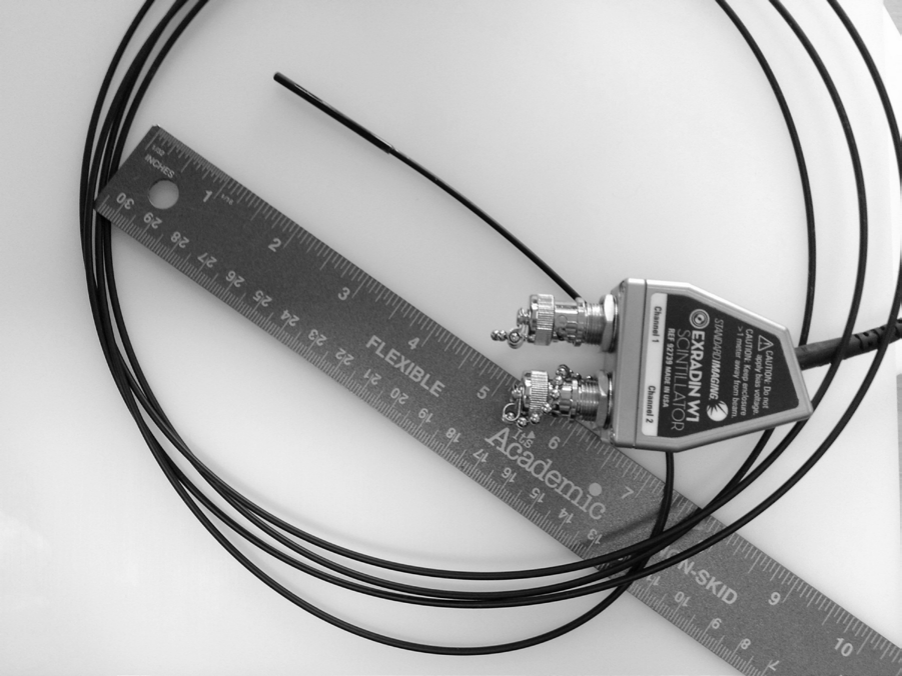
Commercial plastic scintillator dosimeter — the Exradin W1.

The Exradin W1 (Standard Imaging Inc., Middleton, WI) is a commercial miniature plastic scintillation detector (PSD) (see Fig. 2). The sensitive volume of the detector is made of a $2.355\times 10^{-3}{\text{cm}}^{3}$ scintillator, 0.05 cm radius by 0.3 cm long. Calibration is made using the spectral‐based method^(9)^ and was performed using a 6 MV photon beam with a size of $30\times 30{\text{cm}}^{2}$.

Two different ion chambers were also used as the reference detectors. The first was an Exradin A11TW parallel plate ion chamber. Its collecting volume is $0.93\;{\text{cm}}^{3}$ and collector diameter is 2 cm. The A11TW was chosen to measure out‐of‐field dose because of its depth position's accuracy in the buildup region. The second ion chamber selected for this project was a CC04 ion chamber (IBA, Louvain‐La‐Neuve, Belgium). This minichamber has a small collecting volume of $0.04\;{\text{cm}}^{3}$.

### B. Percent depth dose (PDD)

Depth‐dose curves were measured in a $38\times 38\times 37.5\;{\text{cm}}^{3}$ water tank with PSD and the small ion chamber at a SSD of 100 cm. PDD curves were measured for photon beams of 6 and 23 MV. PDDs were measured with a field size of $15\times 15{\text{cm}}^{2}$. The out‐of‐field distance of detectors was 3 cm. Measurements were performed from the surface to a depth of 5 cm and 8 cm for 6 and 23 MV, respectively. Detectors used for these measurements were the PSD and the CC04 ion chamber.

### C. Lateral dose measurements

Lateral dose measurements were performed in a water‐equivalent phantom with length of $60\times 30{\text{cm}}^{2}$ and a thickness of 20 cm. Detectors used for these measurements were the PSD and the parallel‐plate ion chamber.

Measurements with photon beam were performed for both energies available on the linac, 6 and 23 MV, for anterior and posterior field. The isocenter was placed at 10 cm depth in the phantom. Square fields were used with lengths of 10, 20, and 30 cm. Lateral dose measurements were performed at the center of the beam and at 3, 5, 10, and 15 cm out‐of‐field.

In the case of electron beams, both 6 and 18 MeV were used for anterior measurement. Because of the low dose involved in the posterior beam, no measurement was done for this gantry position. The isocenter was placed at surface of the phantom. The electron applicators used were 10, 15, and 20 cm. The lateral positions were at the center of the field, 1 cm inside the field and 1, 3, and 5 cm out‐of‐field.

### D. Treatment planning system (TPS)

The TPS Pinnacle^3^ version 9.6 (Philips Medical Systems, Bothell, WA) was used in this project to compare surface dose out‐of‐field measurement with the dose modeled by a TPS. The water‐equivalent phantom and the water tank were modeled in the TPS by recreating the exact same volume of the phantom or the water tank with the density of water.

## III. RESULTS AND DISCUSSION

### A. Percent depth dose (PDD)

**Figure 3:**
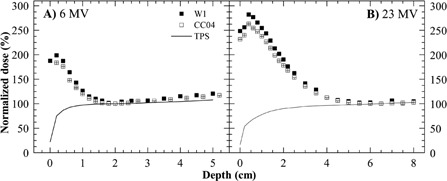
Depth dose for a 15 cm side beam at 3 cm out‐of‐field.

PDD curves for 6 MV photon beam at 3 cm outside the field are shown in Fig. 3. The point of maximum dose measured with the PSD was at 0.2 cm and at 0.4 cm for 6 MV and 23 MV, respectively. Before $D_{\text{max}}$, the TPS doses strongly differ from the measurement (between 25% and 80%). The average difference between the measurements of PSD W1 and IC CC04 is 5% and 9% for 6 MV and 23 MV, respectively.

The behavior of the depth‐dose curves out‐of‐field agrees with the observations of Fraass et al.^(1)^ A change in energy does not change the general behavior of the PDD curve. The dose drops between the surface and the depth of $D_{\text{max}}$ in the center of the field. Beyond this depth, the dose slowly increases with the depth. The same behavior was observed by Starkschall et al.^(10)^ Contrary to the findings of other researchers,^11^, ^12^ for depth dose in the center of the beam, near to the surface, for out‐of‐field measurement, the dose measured by PSD was higher than that measured by IC.

### B. Lateral measurements for photon beams

**Figure 4:**
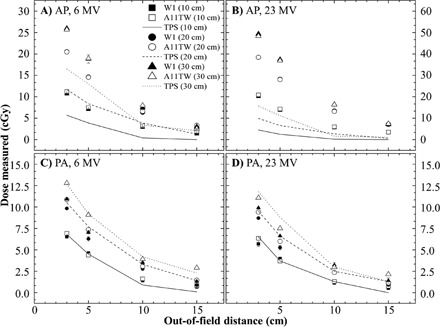
Lateral dose measurement at 0.5 cm depth for anterior ((a) and (b)) and posterior ((c) and (d)) photon beam.

Results of lateral dose measurement are illustrated in Fig. 4. For both energy fields, the widening of field size increases the surface out‐of‐field dose. It is also observed that the increase of dose, for the same widening of field (10 cm), is larger between the two smaller field sizes, as has been reported in literature.^(1)^

For anterior field, the larger different between IC and PSD measurement is under 1% (0.3 cGy) but can reach 12% (0.8 cGy) between dosimeters and TPS. The difference between the ion chamber and the W1 measurements for posterior fields can be explained by the fact that the ion chamber, A11TW, is irradiated from the back to the front, which is not the standard way to use it, due to the impossibility of using it on the opposite side (thickness around 2 cm). The average difference between the results of the W1 and the A11TW at 6 MV are more important than the difference at 23 MV because the results are smaller for the 23 MV beam. For posterior field, the difference between PSD and the ion chamber is larger than anterior fields. The opposite is observed for the TPS calculation.

### C. Lateral measurements for electron beams

**Figure 5:**
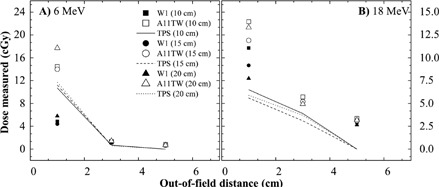
Lateral dose measurement at 0.5 cm depth for anterior electron beam.

Figure 5 shows the lateral dose measurements for electron beams at 6 and 18 MeV. For the 6 MeV beam, there is a large difference between IC and PSD measurement due to the collecting diameter of the IC, which is 2.0 cm. This large difference is not observed with photon beams because the out‐of‐field distance is larger (3 cm). For larger out‐of‐field distance, the differences between IC and PSD are in agreement with results obtained with photon beams. The out‐of‐field dose decreases with the increase of the size of field and this result is observed for both dosimeters.

This study has demonstrated that a PSD can measure a dose as low as 0.51±0.17cGy for photon beams and 0.58±0.20cGy for electron beams, with a difference of 0.2 and 0.1 cGy to measurements made with a parallel‐plate ion chamber. Measurements with PSD at near surface for out‐of‐field dosimetry avoid averaging over a too‐large depth, 1 mm diameter. Electronic equilibrium is easier to reach with PSD due to its small sensitive volume and its water equivalence. PSD shows promise to be a reference dosimeter for out‐of‐field dosimetry.

## ACKNOWLEDGMENTS

This research was supported in part by the Natural Sciences and Engineering Research Council of Canada (NSERC) discovery Grant Nos. 385773.

## COPYRIGHT

This work is licensed under a Creative Commons Attribution 3.0 Unported License.
